# Photoelectrochemical Water Splitting with ITO/WO_3_/BiVO_4_/CoPi Multishell Nanotubes Enabled by a Vacuum and Plasma Soft-Template Synthesis

**DOI:** 10.1021/acsami.2c19868

**Published:** 2023-02-10

**Authors:** Jorge Gil-Rostra, Javier Castillo-Seoane, Qian Guo, Ana Belén Jorge Sobrido, Agustín R. González-Elipe, Ana Borrás

**Affiliations:** † Nanotechnology on Surfaces and Plasma Lab. Instituto de Ciencia de Materiales de Sevilla (CSIC-US). Avenida de Américo Vespucio, 49, 41092 Sevilla, Spain; ‡ School of Engineering and eMaterials Science, Queen Mary University of London, E1 4NS, London, UK

**Keywords:** photoelectrochemistry (PEC), water splitting, oxygen evolution reaction (OER), ITO, WO_3_, BiVO_4_, CoPi, multishell nanotubes (NTs), soft template synthesis, magnetron sputtering

## Abstract

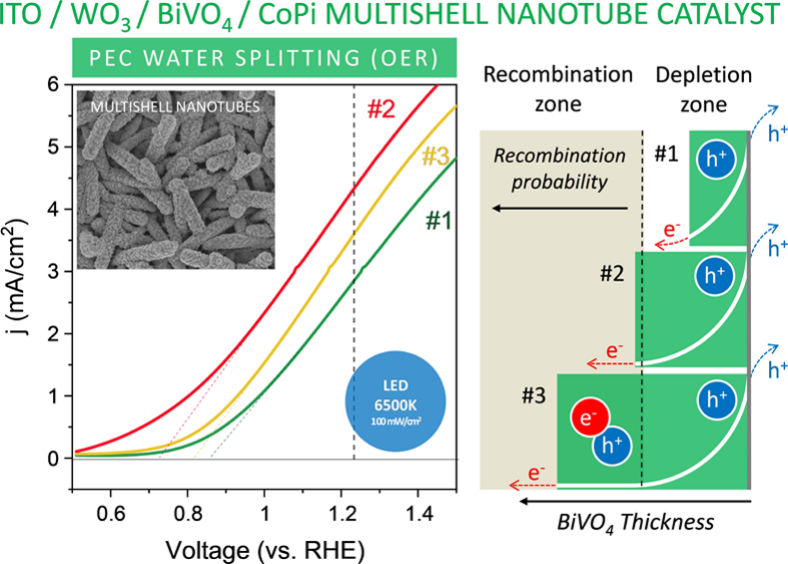

A common approach for the photoelectrochemical (PEC) splitting of water relies on the application of WO_3_ porous electrodes sensitized with BiVO_4_ acting as a visible photoanode semiconductor. In this work, we propose a new architecture of photoelectrodes consisting of supported multishell nanotubes (NTs) fabricated by a soft-template approach. These NTs are formed by a concentric layered structure of indium tin oxide (ITO), WO_3_, and BiVO_4_, together with a final thin layer of cobalt phosphate (CoPi) co-catalyst. The photoelectrode manufacturing procedure is easily implementable at a large scale and successively combines the thermal evaporation of single crystalline organic nanowires (ONWs), the magnetron sputtering deposition of ITO and WO_3_, and the solution dripping and electrochemical deposition of, respectively, BiVO_4_ and CoPi, plus the annealing in air under mild conditions. The obtained NT electrodes depict a large electrochemically active surface and outperform the efficiency of equivalent planar-layered electrodes by more than one order of magnitude. A thorough electrochemical analysis of the electrodes illuminated with blue and solar lights demonstrates that the characteristics of the WO_3_/BiVO_4_ Schottky barrier heterojunction control the NT electrode efficiency, which depended on the BiVO_4_ outer layer thickness and the incorporation of the CoPi electrocatalyst. These results support the high potential of the proposed soft-template methodology for the large-area fabrication of highly efficient multishell ITO/WO_3_/BiVO_4_/CoPi NT electrodes for the PEC splitting of water.

## Introduction

The photoelectrochemical (PEC) splitting of water is considered one of the most important drivers toward the development of energy-sustainable methods for hydrogen production.^1−3^ A widely investigated approach entails the application of efficient visible light semiconductor electrodes for the oxygen evolution reaction (OER).^4−7^ From the materials’ composition point of view, a classical option for this purpose consists of using electrodes formed by the WO_3_ wide bandgap semiconductor sensitized with an external layer of BiVO_4_, this latter acting as a high-performance visible light scavenger semiconductor.^8−12^ Besides enabling the sensitization with visible photons of the solar spectrum, this configuration contributes to overcoming some of the problems found by the implementation of WO_3_ electrodes, namely, a high photohole-photoelectron recombination rate and a considerable corrosion by intermediate peroxo-like species formed during the OER.^13^ Therefore, much effort has been dedicated to the tailored synthesis of layered photoelectrodes through the stacking of these two semiconductors in configurations that maximize the effective interface area with the electrolyte. Among the rich literature on WO_3_ and WO_3_/BiVO_4_ high area photoelectrode structures, we can quote nanoflakes and nanorods prepared by hydrothermal methods,^14,15^ nanowires prepared by flame vapor deposition,^8,16−19^ nanowires decorated by atomic layer deposition,^20^ or helix structures prepared by glancing angle evaporation,^21^ this latter rendering the maximum efficiencies reported so far for this type of bilayer photoelectrode (for a more complete appraisal of preparation methods and available structures, see the reviews in refs (13) and (22)). However, despite the imperious need for large-area scalable methods compatible with common electrocatalyst supports, most reported manufacturing technologies have been tested at a lab scale.^23^ Addressing this challenge, herein, we propose the use of a scalable methodology for the fabrication of substrate-supported nanotubes (NTs) with a multishell nanoarchitecture consisting of an ITO inner layer covered with a WO_3_/BiVO_4_ heterojunction shell bilayer. This concentric architecture around the ITO layer provides a direct drainage pathway for the photoelectrons generated at the WO_3_/BiVO_4_ bilayer, thus minimizing the ohmic resistance to the external circuit. It will be also shown that the arrangement in the form of nanotubes, apart from the obvious enhancement in surface area in comparison with a planar thin film topology, provides a significant scattering of light that contributes to the light absorption capacity of the semiconducting shells.

In addition to the evaluation of the PEC activity of NT electrodes, a critical point for assessment in this work has been to determine the influence of the thickness and morphology of the outer BiVO_4_ shell layer on the OER yield. Recently, Kafizas et al.^24^ and Grigioni et al.,^25^ using transient absorption spectroscopy and planar WO_3_/BiVO_4_ thin film heterojunction electrodes, have shown that photocurrent depends on BiVO_4_ thickness, reaching a maximum yield for a thickness of 75 nm. Herein, we have compared the performance of the NT electrodes for a constant thickness of ITO and WO_3_ layers but variable BiVO_4_ layer thickness. The impedance spectroscopy analysis of the OER photoelectrode’s behavior under blue light excitation^3,26−28^ has provided some clues to understand the charge transfer mechanism at the WO_3_/BiVO_4_ interface and to determine the optimal BiVO_4_ layer thickness. These results have been compared with those obtained with a thin film ITO/WO_3_/BiVO_4_ reference electrode, prepared by the same methodology but formed by the stacking of planar layers and that, therefore, depicted a much lower electrochemically active surface area.^11,29^ The optimized NT topology has been also modified by the addition of a phosphate cobalt co-catalyst (CoPi) to further enhance the OER reaction efficiency.^30−34^ Tested under one sun illumination, this electrode configuration rendered a PEC current of 2.23 mA/cm^2^ for an applied voltage of 1.23 V vs reversible hydrogen electrode (RHE). The obtained results define a set of critical boundary conditions in terms of NT length, semiconductor layer thickness, and co-catalyst load that can be tailored to maximize the efficiency of this type of nanostructured electrodes.

## Photoanode Fabrication Methodology

**Scheme 1:**
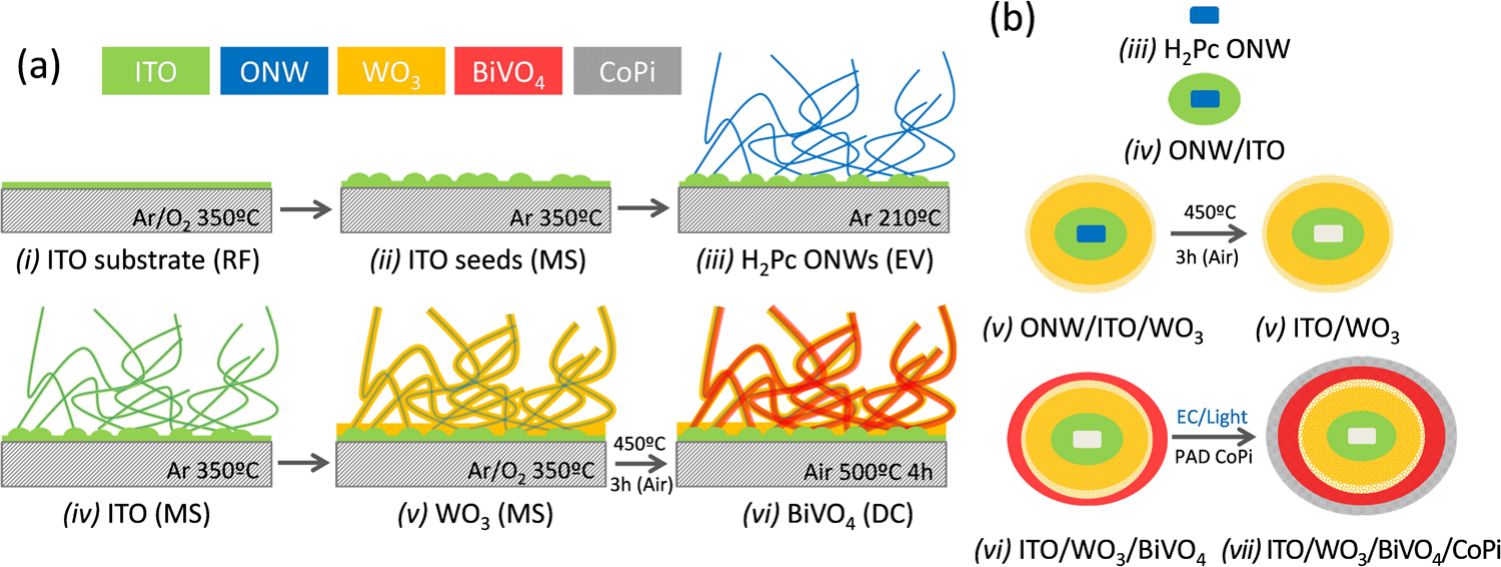
Conceptual Schematic Description (Not to Scale) of the Multistep Synthesis Procedure of the ITO/WO_3_/BiVO_4_ Multishell NT Photoanodes (a) Synthesis steps of the NTs (steps (i)–(vi)) on an ITO glass substrate. (b) Scheme of the evolution of the NT cross section after the successive preparation steps (iii)–(vi) in (a) and after the incorporation of a Co phosphate co-catalyst (step vii) (see also Materials and Methods and Supporting Information S1 section). RF indicates radio-frequency plasma pretreatment; MS, magnetron sputtering; EV, thermal evaporation; and DC, drop casting.

Scheme 1a presents a conceptual representation of the synthesis steps required to produce the NT electrodes. The method is large-area compatible and, regarding the incorporation of the ITO and WO_3_ layers, can be carried out in a single vacuum deposition reactor (i.e., following a one-reactor approach).^35^ The fundamentals of the procedure rely on the use of small-molecule single-crystalline organic nanowires (ONWs) as soft-template 1D scaffolds, which can be fabricated and removed upon mild heating in vacuum or air. Although this work is the first example showing the application of this methodology for the fabrication of electrodes formed by a complex multilayered architecture (i.e., ITO/WO_3_/BiVO_4_), it has a general character and has been recently applied to other oxide materials and applications.^36,37^ In steps (i)–(iii), ONWs are formed by the self-assembly of pi-stacked small molecules such as porphyrins, perylenes, and phthalocyanines. The ONWs grow under defined vacuum and temperature conditions by evaporation of the molecules on the desired support with previously deposited ITO nuclei. In the present work, the ONWs were made by the stacking of commercially available H_2_-phthalocyanine (H_2_Pc) molecules. A detailed description of the mechanisms behind this fabrication process can be found elsewhere.^38,39^ A singular advantage of this procedure is its compatibility with a large variety of materials and substrates including polymers, soda lime glass, metallic meshes, ceramics, and fabrics or paper, among others. In a subsequent step (iv), an ITO layer is deposited around the ONWs. For this step, we follow the procedure described in ref (35) where we have reported the fabrication by magnetron sputtering (MS) of ITO nanotubes (NTs) on a flat ITO substrate. MS is a well-established industrially scalable technique usually employed for the fabrication of compact thin films and, more recently, also for porous structures of WO_3_ electrodes^40^ and WO_3_/BiVO_4_ photoelectrodes,^41,42^ the former under an oblique angle configuration. The versatility of the MS procedure enables a conformal deposition of the ITO and then the WO_3_ layers (step (v)) in the same reactor (see the Materials and Methods and Supporting Information sections for details). In step (vi), the multishell architecture is covered with a third layer of BiVO_4_ deposited by drop casting. The annealing removal of the organic scaffold rendered substrate-supported multishell NT electrodes, which have been extensively characterized and photoelectrochemically tested. Scheme 1b shows a cross-sectional representation of the sequential growth of the NT shell structure (steps (iii) to (vi)). It highlights that the NTs integrate a transparent conductive ITO core covered by concentric WO_3_ and BiVO_4_ layers. This configuration ensures a high surface area, the adjustment of the WO_3_ and BiVO_4_ semiconductor bands in line with the expected electronic behavior of this heterojunction,^9,13,16,22,30,42,43^ and a minimization of the photoelectron path required to reach the ITO draining electron layer. As a final step in the preparation procedure, a very thin layer of cobalt phosphate (CoPi) is added by a photoelectrochemical-activated deposition technique (c.f. Scheme 1b (vii)).

It is noteworthy that the synthesis procedure along the steps (i)–(vii) is quite versatile and permits a fine control of the electrode microstructure by, for example, varying the areal density of NTs (i.e., changing the ITO nucleation sites in step (ii)), the length of NTs (i.e., controlling the length of the ONWs through the adjustment of the evaporation time of the organic molecules in step (iii)), or the thickness of the different layers that constitute the multishell structure (i.e., modifying the MS deposition conditions in steps (iv) and (v)). This versatility would enable the optimization of electrode performance. In this work, besides analyzing the performance of a conventional PEC electrode configuration, we have specifically addressed the dependence of PEC efficiency on the thickness of the BiVO_4_ layer prepared in step (vi).

## Results and Discussion

### Characterization and Optical Properties of ITO/WO_3_/BiVO_4_ NT Electrodes

**Figure 1:**
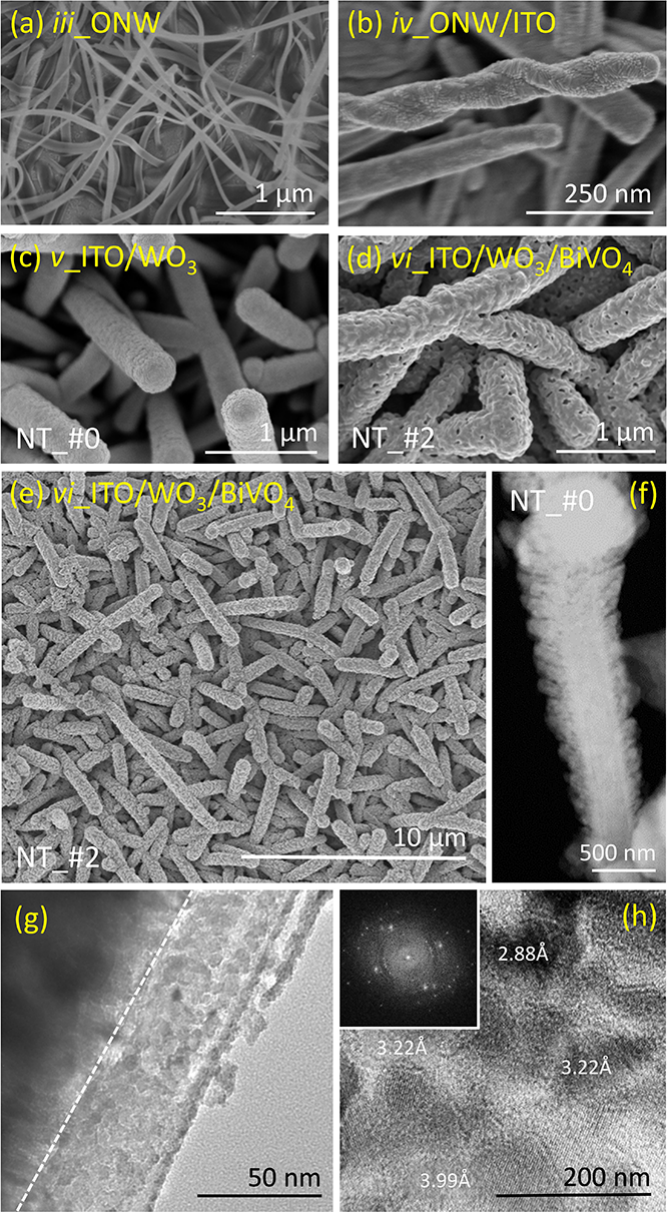
(a–e) Selected top view SEM micrographs at various magnification scales of the hollow multishell NT samples at different stages of their manufacturing process according to Scheme 1. (f–h) Analysis of a single NT retrieved from sample NT_#0: (f) HAADF-STEM image, (g) bright-field TEM image, and (h) HREM-diffraction patterns. Please note that labels indicate the different steps (i)–(vi) detailed in Scheme 1.

In this section, we present a thorough description of the morphology, crystallinity, chemical composition, and optical properties of the NT samples. Figure 1 shows a series of SEM micrographs illustrating the evolution of the ONWs and hollow NTs throughout the successive steps of the preparation protocol depicted in Scheme 1. These images are top-view micrographs at different magnifications of the ONWs scaffold (Figure 1a), first covered by the ITO shell (Figure 1b) and then by the WO_3_ shell (Figure 1c). Figure 1c also shows the effect of the annealing treatment to completely remove the organic core formed by the said ONWs. It will be demonstrated below that this thermal treatment also ensures the required stoichiometry and crystallization of the WO_3_ shell. Figure 1c presents the image of a sample with a shell structure ITO/WO_3_, hereafter labeled NT_#0 (cf. Materials and Methods section). Figure 1d,e shows selected SEM micrographs at two magnification scales taken after deposition and annealing of the BiVO_4_ semiconductor layer (samples ITO/WO_3_/BiVO_4_, which are labeled NT_#1, NT_#2, and NT_#3, depending on the equivalent thickness of the BiVO_4_ layer). The lower magnification image of sample NT_#2 in Figure 1e clearly shows that the electrodes are formed by supported NTs with an average surface density of 5–7 NTs/μm^2^ and a maximum length of 10 μm. It should be noted that the length and thickness of NTs can be varied through the adjustment of the fabrication conditions of the ONWs (for the density and length, see refs (27) and (28)) and the deposition time of the shell layers to effectively control their thickness. The choice of an average length in the order micrometers and a relatively low areal density of ONWs was a compromise to maximize the conformality and thickness homogeneity of the shell layers prepared by MS along the length of the NTs. The comparison among Figure 1a–d reveals that the twisted and flexible microstructure of the pristine organic ONW templates (Figure 1a) transforms in a rather vertical arrangement of NTs when the ONWs become MS coated by ITO and then WO_3_ (Figure 1b,c). This evolution has been previously reported for TiO_2_ and ZnO NTs fabricated by the same soft template approach using plasma-enhanced chemical vapor deposition. It has been attributed to the vertical alignment of the NWs under the effect of the electrical field lines of the plasma sheath and the increase of the rigidity of the NTs after coating with the metal oxide layer(s).^44^

Figure 1f–h shows a detailed electron microscopy analysis (i.e., including HAADF-STEM (f), bright-field TEM (g) images, and an HREM-diffraction pattern (h)) of a single nanotube retrieved from sample NT_#0 (Figure 1c). This analysis shows the hollow space left in the interior of the NTs after the annealing removal of the ONW core (Scheme 1a,b, step (v)). Figure 1f shows that the empty core in the center of the NTs is homogeneously surrounded by the ITO and WO_3_ layers. It also reveals that the WO_3_ outer shell was structured in the form of nanocolumns perpendicular to the core axis. This microstructure will likely provide a high surface area and therefore contribute to increasing the PEC activity of the electrodes. On the other hand, the bright-field image in Figure 1g demonstrates that the ITO/WO_3_ interface is sharp and well-defined, while the high-resolution electron micrograph (HREM) in Figure 1h proves the polycrystalline character of the grains and their random orientation. In this HREM image and the inset showing the electron diffraction diagram, it is also possible to recognize the diffraction features and interplanar distances typical of the crystalline planes of WO_3_. It is also apparent in Figure 1f that the NT width slightly varies from the tip to the side through which it anchors onto the ITO substrate. This particular shape of NTs is a consequence of the MS growth mechanism during the deposition onto 1D scaffolds.^35^ Depending on conditions, some of the MS-deposited particles may be ballistic and undergo shadowing effects during their trajectory from the target up to their landing position on the surface, preferentially accumulating at the outer parts of nanostructures.^45^ To minimize this effect and therefore achieve a more homogeneous distribution of material along the NT length, deposition conditions should favor the particle randomization through collisions with the plasma gas molecules (essentially, this is achieved by increasing the plasma gas pressure and the distance target–substrate during deposition).^46^

**Figure 2:**
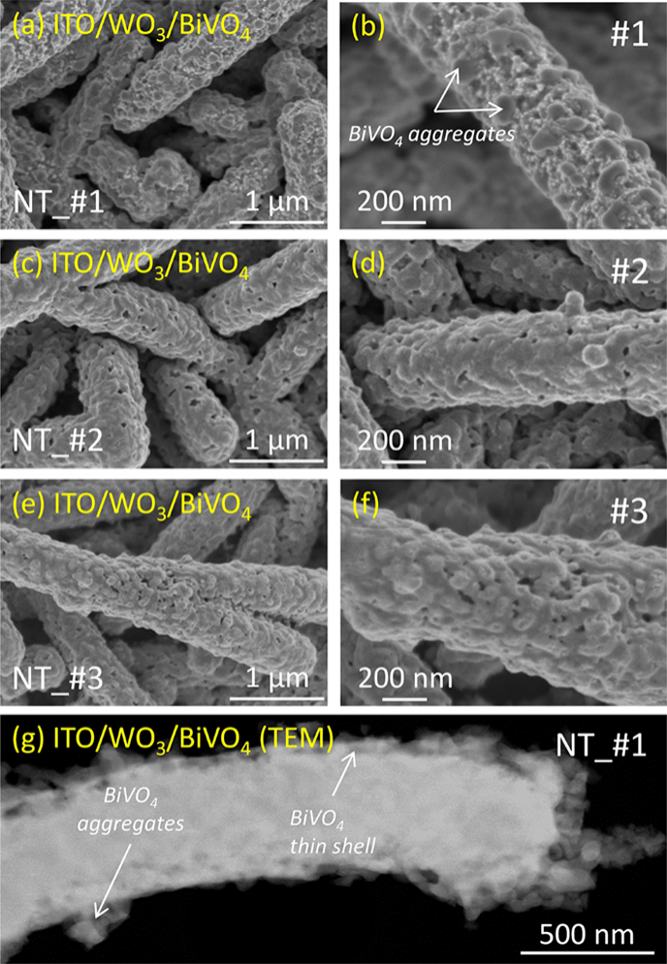
(a–f) Top view SEM micrographs at two different magnifications (1 μm and 200 nm scale) of the multishell NTs, for increasing amounts of BiVO_4_ from samples NT_#1, NT_#2, to NT_#3, as labeled. (g) HAADF-STEM image of a single NT taken from sample NT_#1.

The next step of the photoanode manufacturing process consisted of the deposition of BiVO_4_ by drop-casting (Scheme 1 (a), step (vi)). Figure 2a–e shows characteristic SEM micrographs, at low and high magnifications, of the NT structure of samples NT_#1, NT_#2, and NT_#3. Figure 2g presents a HAADF-STEM image of an individual NT taken from sample NT_#1. This image proves that the microstructure of the BiVO_4_ layer is formed by grains covering the NT outer surface (see Figure S1 in the Supporting Information section for better defined cross-sectional views of samples NT_#1 and NT_#3 obtained by processing the single nanotubes by a focused ion beam (FIB)). As expected, the average NT width increases with the amount of BiVO_4_ (i.e., with the number of drop casting steps), reaching an average width higher than 500 nm in sample NT_#3. Micrographs in Figure 2a–f demonstrate a progressive homogenization of the BiVO_4_ layer from sample NT_#1 to sample NT_#3. Thus, in sample NT_#1, BiVO_4_ forms a very thin layer (thickness in the order of a few tenths of a nanometer), which fills the porous structure of the WO_3_ layer beneath, together with small imperfectly coalesced aggregates. EDX maps in Figure S1 confirm that BiVO_4_ spreads relatively well onto WO_3_ in sample NT_#1, while in samples NT_#2 and NT_#3, this compound forms large aggregates, which are partially connected in a kind of granular shell (Figure 2c–f).

Although an accurate estimation of the NT thickness is hampered by the inherent random distribution of sizes of these structures, a statistical analysis of the SEM images taken at medium magnifications at selected representative areas of samples (see data gathered in Table S1 of the Supporting Information) gives the following average values of NT width: 438 ± 64 nm (sample NT_#0), 483 ± 35 nm (sample NT_#1), 568 ± 78 nm (sample NT_#2), and 582 ± 48 nm (sample NT_#3). These measurements render approximate BiVO_4_ layer thicknesses of 30, 65, and 85 nm for samples NT_#1, NT_#2, and NT_#3, respectively. For comparison, the mean thickness values of the WO_3_ and BiVO_4_ layers stacked in sample TF, estimated from the cross-sectional micrograph in Figure S2, were 550 and 370 nm, respectively.

**Figure 3:**
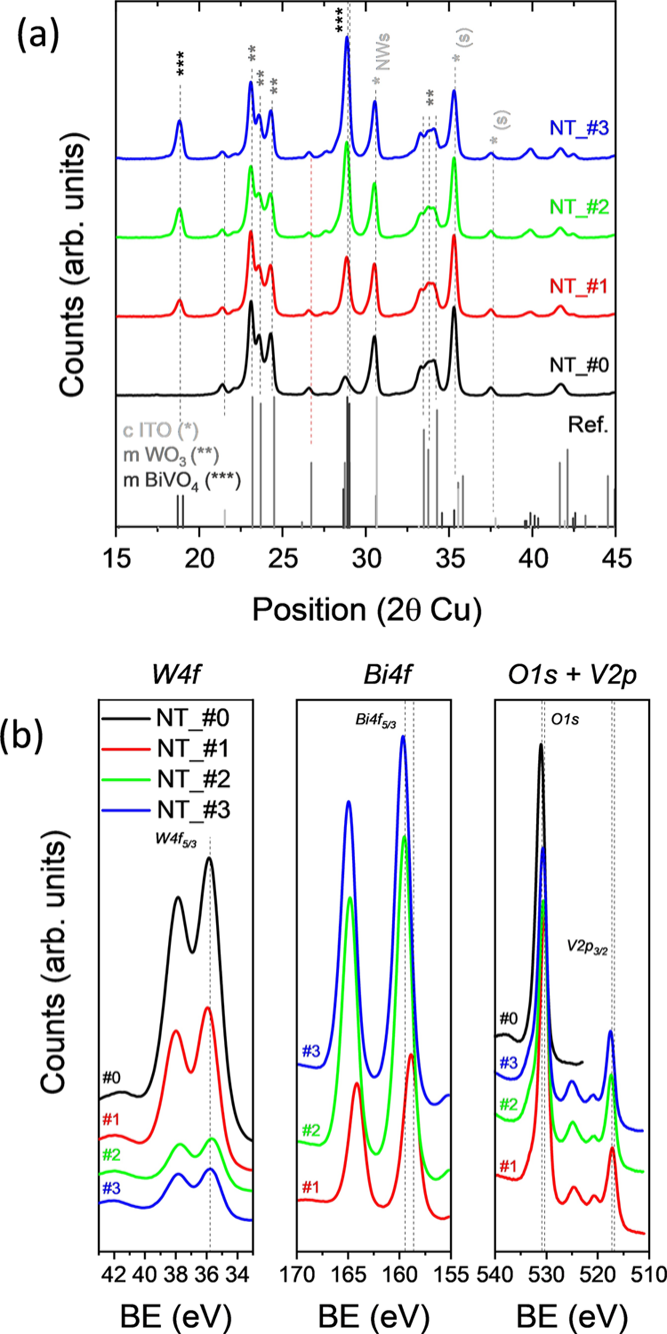
(a) XRD diagrams for samples NT_#0 to NT_#3. Peaks attributed to the cubic structure of In_2_O_3_/SnO_2_ (*),^47^ the monoclinic structure of WO_3_ (**),^48^ and the monoclinic structure of BiVO_4_ (***)^49^ can be identified in the diagrams. (b) W4f, Bi4f, and O1s/V2p high-resolution XPS spectra of samples NT_#0, NT_#1, NT_#2, and NT_#3.

The crystal structure of WO_3_ and BiVO_4_ semiconductors is of paramount importance for a proper operation of WO_3_/BiVO_4_ heterojunction photocells.^8−12,16,22,42,43^ An analysis of the crystallinity of the NT electrodes by XRD rendered the diagrams in Figure 3a for samples NT_#0, NT_#1, NT_#2, and NT_#3. These diagrams depict the typical peaks of well-crystallized ITO (cubic structure), WO_3_ (monoclinic structure), and BiVO_3_ (monoclinic structure).^47−49^

The deposition of BiVO_4_ by drop-casting and the chemical nature of the surface of NTs were investigated by XPS and EDX. The comparison of low-resolution XPS spectra reported in Figure S3 shows a progressive decrease of the W4f/Bi4f intensity ratio when passing from sample NT_#0 to samples NT_#1 and NT_#2, while differences in this parameter were smaller when comparing samples NT_#2 and NT_#3. This evolution confirms the aforementioned SEM observations in the sense that the coverage of BiVO_4_ onto the WO_3_ shell is rather conformal in samples NT_#1 and NT_#2, while in sample NT_#3, there is a certain agglomeration of this compound in the form of relatively big clusters. The fact that the W4f signal is still visible in samples NT_#1, NT_#2, and even NT_#3 also indicates that some zones of WO_3_ are not completely covered by BiVO_4_. Meanwhile, the series of high-resolution XPS spectra gathered in Figure 3b, besides supporting this assessment about coverage based on the low-resolution spectra, confirm that W^6+^, Bi^3+^, and V^5+^ are the oxidation states of these elements in the examined samples (B.E.s W4f (36 eV), Bi4f (159 eV), and V2p (517 eV)). In this regard, a deeper analysis of the O1s high-resolution XPS spectra (see Supporting Information, Figure S3) leads to the attribution of its components to lattice oxygens in WO_3_ (O-W) for sample NT_#0 and to an oxygen coordination environment in BiVO_4_ (O-V) for samples NT_#1–#3, respectively, which confirms the previous NTs’ surface composition assessment.

In parallel to the changes outlined above on morphology and surface chemistry, samples underwent color changes after each processing step. In particular, a white-yellow transformation was observed between steps (v) and (vi). This color change must respond to the different diffuse reflectance spectra of samples NT_#0 (with only ITO and WO_3_ layers) and NT_#1 to NT_#3, in these latter after the incorporation of BiVO_4_ (see Supporting Information, Figure S4). Most remarkable in these spectra is the shift in the absorption edge from sample NT_#0 (approximately 2.7 eV) to samples NT_#1–#3 (approximately 2.4 eV for the three samples). This shift proves that the incorporation of BiVO_4_ produces the yellow coloration of samples due to the capacity of this semiconductor to absorb visible light in the wavelength interval comprised between approximately 400 and 450 nm. Most PEC experiments described next were done illuminating the photoanodes with photons with a wavelength within this energy window, i.e., to selectively induce the excitation of the BiVO_4_ semiconductor.

It will be shown in the next section that electrode samples NT_#1–#3 depict PEC efficiencies that are orders of magnitude higher than that of the reference thin film sample (TF). Such an improvement in photocurrent will likely depend on the increase in the surface area provided by the NT structure of samples NT_#1–#3, although a certain enhancement in light scattering due to the random arrangement of NTs may also contribute to increasing the photocurrents^3^ (we have recently reported on a significant light scattering for NT structures of ITO).^35^ The critical role of the high surface area of samples NT_#0–#3 working as photoanodes and its effect on the photocell efficiency was confirmed by determining their effective electrochemically active surface area (ECSA).^50−54^ For this purpose, we applied the double-layer capacitance method to deduce capacitance values from the slope of current density vs scan rate plots (see Supporting Information, Figure S5). As expected, the obtained values for the multishell NT electrodes (see Figure S5) were almost two orders of magnitude higher than for the reference TF, thus confirming the importance of ECSA for an effective control of photoefficiency. Interestingly, it was also found that the incorporation of BiVO_4_ did not significantly modify the capacitance values of NT samples, thus indicating that samples NT_#0 to NT_#3 exhibited rather similar ECSA values.

### PEC Activity of Thin Film, Multishell NTs, and CoPi-Modified Electrodes

Voltammetric and chronoamperometric tests were carried out with the NT and NT-CoPi photoelectrodes to assess their PEC activity in comparison with that of the TF samples. Most tests were carried out upon illumination with a 6500 K LED, although experiments were also performed under illumination with a solar simulator to determine the response of the most efficient NT electrodes according to standardized procedures.^55^

**Figure 4:**
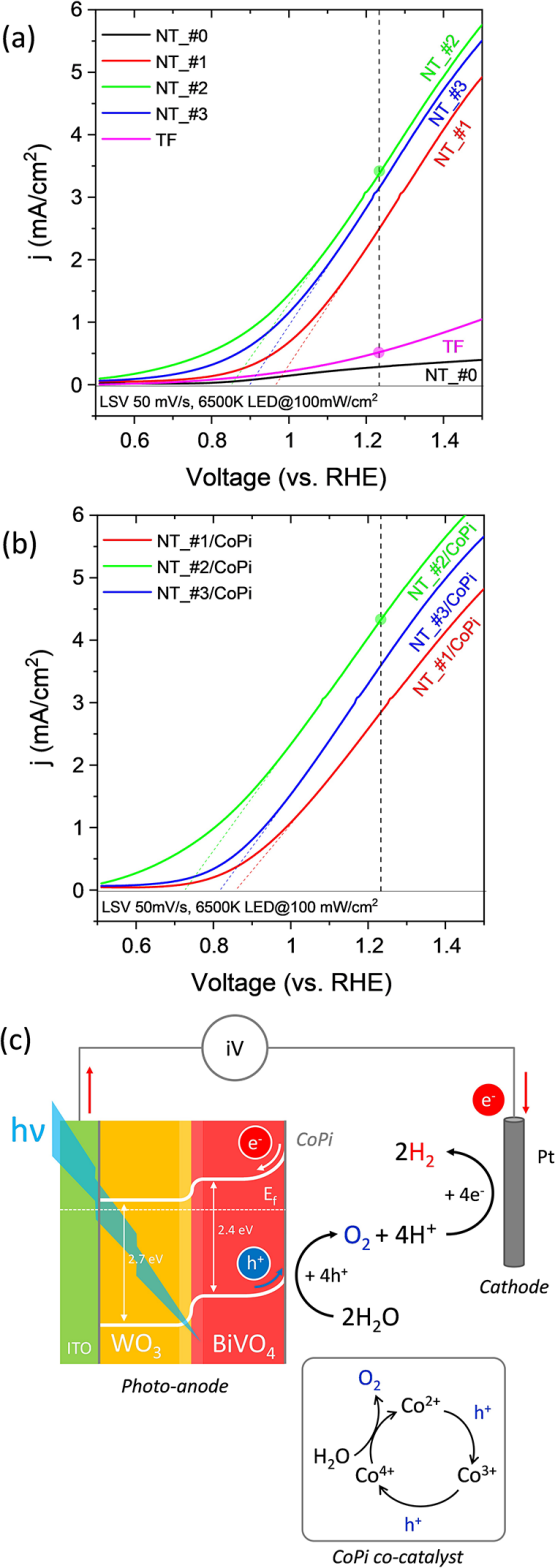
LSV analysis of NT electrodes under LED 6500 K blue light (420–475 nm) illumination. (a) LSV diagrams under illumination with LED 6500 K blue light for multishell NTs (i.e., NT_#0–#3) and TF electrodes. (b) LSV diagrams under illumination with LED 6500 K blue light for NT_#1/CoPi, NT_#2/CoPi, and NT_#3/CoPi electrodes. (c) Band diagram describing the fate of photoelectrons and photoholes upon light absorption by the BiVO_4_ semiconductor. Inset: schematic description of the catalytic mechanism of the CoPi co-catalyst to activate the OER.

The PEC performance of NTs and TF electrodes was first assessed by linear sweep voltammetry (LSV) analysis. Figure 4a shows the LSV curves measured for samples NT_#0–#3 and TF under illumination with the blue light of a 6500 K LED (see Materials and Methods section) for a light flux power of 100 mW cm^–2^. Samples NT_#0 and TF present a much lower PEC efficiency than samples NT_#1 to NT_#3. This difference must be attributed to the lack of the sensitizing BiVO_4_ semiconductor layer in sample NT_#0 and the significantly lower ECSA and light scattering capacity in the reference TF sample. Meanwhile, a comparison among samples NT_#1–_#3 shows that efficiency is maximum for sample NT_#2. A more complete LSV analysis of the PEC response of multishell NT electrodes as a function of the light flux (provided by a high-intensity LED 6500 K light source) is reported in Supporting Information, Figure S6. This complementary analysis revealed that current density did not saturate, even for light intensities as high as 300 mW/cm^2^, and that for this high photon flux, the PEC currents determined for samples TF and NT_#0 were comparatively very small.

The observed photocurrents of samples NT_#1–#3 are linked to the visible absorption capacity of the BiVO_4_ layer. According to common models of photoexcitation of sensitizing semiconductors,^8−12^ photon absorption by BiVO_4_ produces electron–hole pairs. As shown in the scheme in Figure 4c, the Schottky band bending of the BiVO_4_ semiconductor at the water–photoelectrode interface promotes the migration of the photoholes to the surface where they react with water to yield a series of very reactive intermediate species (e.g., OH*, OOH*, O*),^56,57^ which upon subsequent reactions lead to the generation of O_2_. Meanwhile, the photoelectrons promoted to the conduction band diffuse to the conduction band of WO_3_, then, through the ITO conductive layer, to the external circuit, and eventually to the cathode where they chemically reduce water to hydrogen. The high efficiency found for this process with the NT electrodes supports a good matching at the heterojunction between the conduction bands of the two semiconductors (see Figure 4c). In addition, the particular architecture of the NT photoanodes makes that photoelectrons only need to diffuse through the WO_3_ shell thickness to find the ITO draining layer, i.e., through a short path, therefore reducing the ohmic resistance associated with the transport of electrons. Interestingly, equivalent experiments with green and red LEDs (emissions centered at 520 and 635 nm, respectively) did not produce any significant PEC reaction, in agreement with the negligible photon absorption coefficient of BiVO_4_ at these wavelengths (data not shown).

The OER efficiency further increased when the NTs were decorated with the CoPi catalyst applied using a rather standard procedure. The LSV plots recorded in Figure 4b for samples NT_#1/CoPi to NT_#3/CoPi reveal an increase in current density for these three samples compared to the equivalent ones without the OER co-catalyst (i.e., NT_#1–#3). It is known that the OER catalytic activity of cobalt catalysts involves the formation at high positive voltages (V > 1.23 V vs RHE) of Co^3+^ and Co^4+^ intermediate species, the latter in the form of CoO(OH) units (see the scheme in the inset of Figure 4).^58^ Interestingly, the plots in Figure 4b reproduce the order in efficiency found for the NT electrodes without catalyst, i.e., #2/CoPi > #3/CoPi > #1/CoPi.

**Figure 5:**
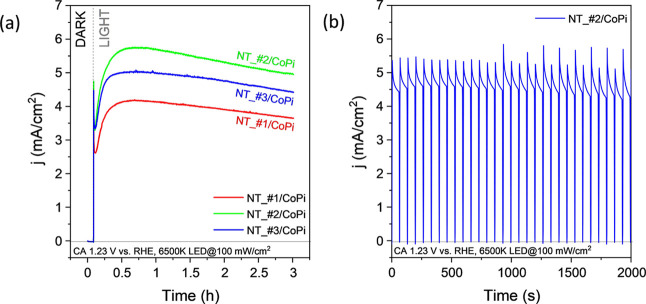
Chronoamperometry tests of NT electrodes with CoPi co-catalysts. (a) CA diagrams at 1.23 V vs RHE and a LED 6500 K light source power flux of 100 mW/cm^2^ recorded during a period of 3 h for samples NT_#1/CoPi, NT_#2/CoPi, and NT_#3CoPi. (b) CA curves obtained under the same experimental conditions for sample NT_#2/CoPi while chopping the illumination approximately every 60 s.

The order in efficiency deduced from the LSV analysis (i.e., samples #2 > #3 > #1) was confirmed by long-term chronoamperometry tests (CA). Figure 5a shows the CA curves recorded as a function of time for an applied voltage of 1.23 V vs RHE. Please note that these results correspond to the NT electrodes with the CoPi co-catalyst, although the same trend was also obtained without the co-catalyst. According to these curves, the photocurrent intensity depicts an initial increment for the first 30 min, followed by a smooth decrease in 3 h. A similar behavior^59^ has been attributed to the effective initial formation of Co^3+^ and Co^4+^ species during the first activation period followed by a subsequent stabilization process.^59^

The summary of average photocurrent values gathered in Supporting Information, Figure S7, determined by the LSV and CA experiments in Figures 4 and 5 for the NT electrodes with and without CoPi, confirms that samples NT_#2 and NT_#2/CoPi depict maximum efficiencies. Interestingly, when the used samples were taken out of the liquid medium, dried, and stored for 3 months to then perform a new experiment, the current density vs time trend depicted in Figure 5a was completely reproducible. This result confirms the stability and robustness of the NT electrodes and their long-lasting stability. The photon-assisted character of the electrolysis and the long-term stability of this process was confirmed by complementary CA tests where the light provided by the 6500 K LED was systematically chopped at small time intervals. Figure 5b illustrates the results of an experiment carried out with sample NT_#2/CoPi. This figure shows that current is illumination-dependent and that current intensity after multiple chopping processes always recovers the value upon illumination without any significant decrease.

**Figure 6:**
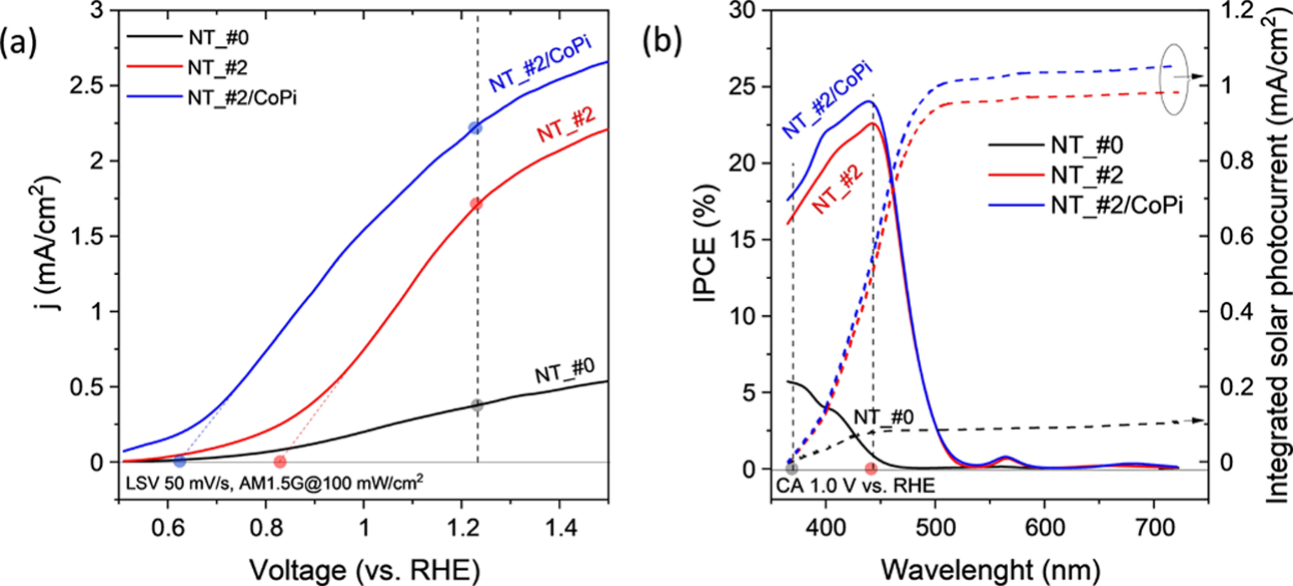
(a) Electrode sensitization under illumination with a standard 1 sun solar simulator (AM1.5 G). LSV plots corresponding to samples NT_#0, NT_#2, and NT_#2/CoPi. (b) IPCE curves, and integrated solar photocurrent (AM1.5 G) (dashed lines) taken at 1.0 V (vs RHE) for these same electrode samples.

The analysis of the CA plots in Figure 5b can be used to estimate transient times for the photo-hole migration through the BiVO_4_ semiconductor. According to the works in refs 58 and 60 the analysis of the initial transient decay up to stabilization of the current curve (see Supporting Information, Figure S8) can provide an estimate of the average diffusion time of photoholes in their path toward the surface. The diffusion time (τ) can be obtained through the following expressions:^60,61^
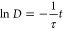
1
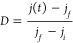
2where *D* can be determined experimentally as indicated in Figure S8. Calculated values of the diffusion time (τ) amounted to 9.5 μs for sample NT_#0 (note however the small current generated with this electrode, c.f., Figure 5a and Figure 6a) and 12.8 μs for the three multishell NT_#1/CoPi–#3/CoPi electrodes. These values are in good agreement with those previously reported for other nanostructured electrodes of these materials.^22,60,61^ The similar photohole diffusion values found for the three multishell NT electrodes suggest that the observed differences in efficiency must involve additional factors, such as differences in reaction kinetics at the electrode interface or in other charge transfer phenomena that will be discussed in the next section.

LSV diagrams for electrodes NT_#2, NT_#2/CoPi, and NT_#0 were also recorded under 1 sun illumination with a standard AM1.5 G solar simulator lamp (see details in the Methods section). This experiment aimed at properly comparing according to standard methods^55^ the photocurrents rendered by NT electrodes with those reported in the literature for other electrode configurations.^8−12^ According to Figure 6a, upon illumination with the complete solar light spectrum, the electrode NT_#0 provides a low current density, although the intensity was significantly smaller than that measured with electrode NT_#2. Figure 6a also shows a net increase in efficiency when incorporating the CoPi co-catalyst (i.e., for sample NT_#2/CoPi), photocurrent amounting to a maximum of 2.23 mA/cm^2^ at 1.23 V vs RHE. Although this value is still far from the maximum values reported for other nanostructures of the same materials,^13,21,41^ it is in the order of recent efficiencies reported for nanostructures resembling those used in the present work, though prepared by other methods.^22^ We expect that the versatility of the template methodology applied in the present work and the basic information about semiconductor heterostructures deduced here will permit further improvements in efficiency. It is also noteworthy that the threshold potential for the OER, determined by a simple extrapolation of the *j* vs *V* curve, presented a remarkably low value of 0.63 V for this electrode. On the other hand, the incident photon to current conversion efficiency (IPCE) curves and the integrated solar photocurrent curves (AM1.5 G) gathered in Figure 6b, taken at a voltage of 1.0 V vs RHE reference electrode, confirm that samples NT_#2 and NT_#2/CoPi are effectively sensitized with λ < 500 nm photons, while sample NT_#0 only presents a photoresponse for λ < 450 nm photons.

### Electrode Morphology and Charge Transfer Processes at the NT Electrode–Electrolyte Interface

To further account for the impact of NT morphology on PEC cell efficiency, we have characterized the cell electrical behavior by electrochemical impedance spectroscopy (EIS) and analyzed the charge transfer kinetics at the interface using Tafel analysis, either with or without the CoPi co-catalyst. As a result, we have gained a better understanding of the main factors contributing to the high efficiency of the NT electrodes and, in this way, unraveled the mechanisms promoting samples NT_#2 and NT_#2/CoPi as the most efficient photoelectrodes.

**Figure 7:**
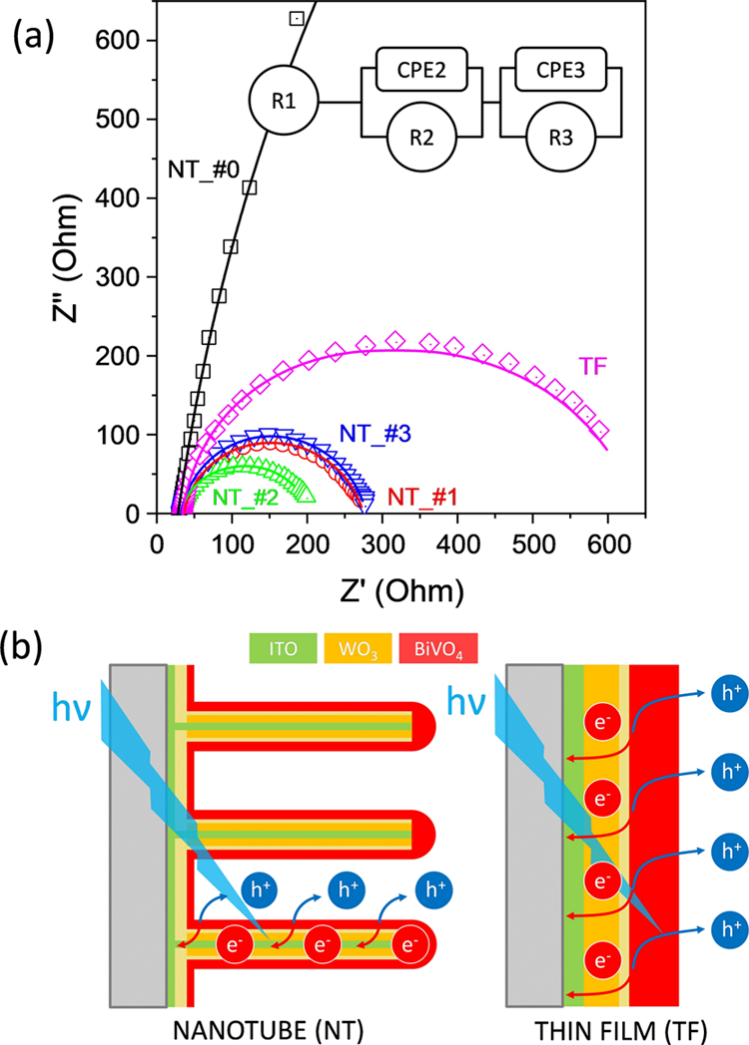
(a) Nyquist plots for samples NT_#0–#3 and TF recorded under 6500 K LED illumination with an intensity of 100 mW/cm^2^ and an applied voltage of 1.23 V vs RHE. Inset: scheme of the equivalent circuit. (b) Scheme of the NT and TF electrode nanostructures and the involved PEC process, highlighting the operative advantages of the ITO/WO_3_/BiVO_4_ nanotube structure.

Nyquist plots obtained by EIS are reported in Figure 7a. These plots can be fitted by assigning specific values to the electrical elements of the equivalent circuit included as an insert in this figure. Similar types of circuits have been proposed to account for the charge transfer behavior in other WO_3_/BiVO_4_ nanostructured heterojunction electrodes.^26−28^

The equivalent circuit incorporates three elements: (i) element 1 characterized by a resistance *R*1, which is attributed to the resistance of the electrolyte solution, wires, clips and ITO substrate layers; (ii) element 2, consisting of a resistance *R*2 and a constant phase element CPE2 that respond to the effect of the interfaces created between the semiconductor oxides integrated into the NTs and TF samples; (iii) element 3, including a resistance *R*3 and a constant phase element CPE3 that account for the charge transfer at the BiVO_4_/electrolyte interface upon light illumination. Phase constant elements instead of pure capacitance elements are used in the scheme because of the non-linearity effects appearing at heterogeneous and nanostructured surfaces and interfaces.^26,62,63^ The impedance associated with the CPEs can be formulated according to
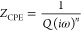
3where *Q* is a CPE parameter expressed in F·s, *n* is a number between 1 and 0, and ω is the angular frequency. The set of Nyquist plots reported in Figure 7 has been fitted using the parameters summarized in Table 1 (for the quality of fitting results see the continuous lines in Figure 7a). These parameters encompass calculated values of resistance *R*, *Q* (directly related to the characteristic non-ideal capacitance of the said element), and an ideality factor (*n*) for each constant phase element.

**Table 1:** Fitting Parameters Reproducing the Shape of the Nyquist Plots Recorded at 1.23 V vs RHE under LED 6500 K Illumination (100 mW/cm^2^)

		NT_#0	NT_#1	NT_#2	NT_#3	TF
	*R*1 (Ohm)	28.2	35.3	31.75	32.3	33.6
	*R*2 (Ohm)	2011.4	204.9	150.4	147.7	429.5
	*R*3 (Ohm)	2530.5	34.7	22.5	98.3	175.4
CPE2	*Q*2 (F·s/10^–4^)	2.4	3.0	4.7	5.1	5.0
	n2	1.00	0.82	0.79	0.85	0.81
CPE3	*Q*3 (F·s/10^–4^)	2.3	3.8	3.9	3.2	2.0
	n3	0.86	1.01	1.01	0.93	0.99

The *R*1 parameter is rather similar for the studied samples, confirming its attribution to the overall electrical resistance of electrolyte plus other ohmic elements in the external circuit between the photoanode and cathode.^26^ On the other hand, *R*2, *R*3, and their sum are more than one order of magnitude higher for electrode NT_#0 than for the other NTs and TF electrodes, proving that the incorporation of a BiVO_4_ outer layer is responsible for the photogeneration of charge upon blue light excitation. CPE2 and CPE3 are also higher for these electrodes than for electrode NT_#0. It is also remarkable that *R*2 + *R*3 values for NT electrodes are much smaller than for TF electrodes, supporting the dependence of the PEC activity on the effective ECSA available in each case. In line with the proposal in previous sections, an additional factor contributing to reducing the value of *R*2 + *R*3 for the NT electrodes must be the straightforward collection of photogenerated electrodes by the inner ITO layer extending along the whole nanotube structure. In summary, as schematized in Figure 7b, we claim that the core–shell ITO/WO_3_/BiVO_4_ nanostructure of the NT electrodes offers simultaneously a high electrochemical surface for the OER and minimum electrical resistance losses due to its particular core–shell configuration. The scattering of light within the NT structure is another factor contributing to the increase in the photocurrent.^35^

**Figure 8:**
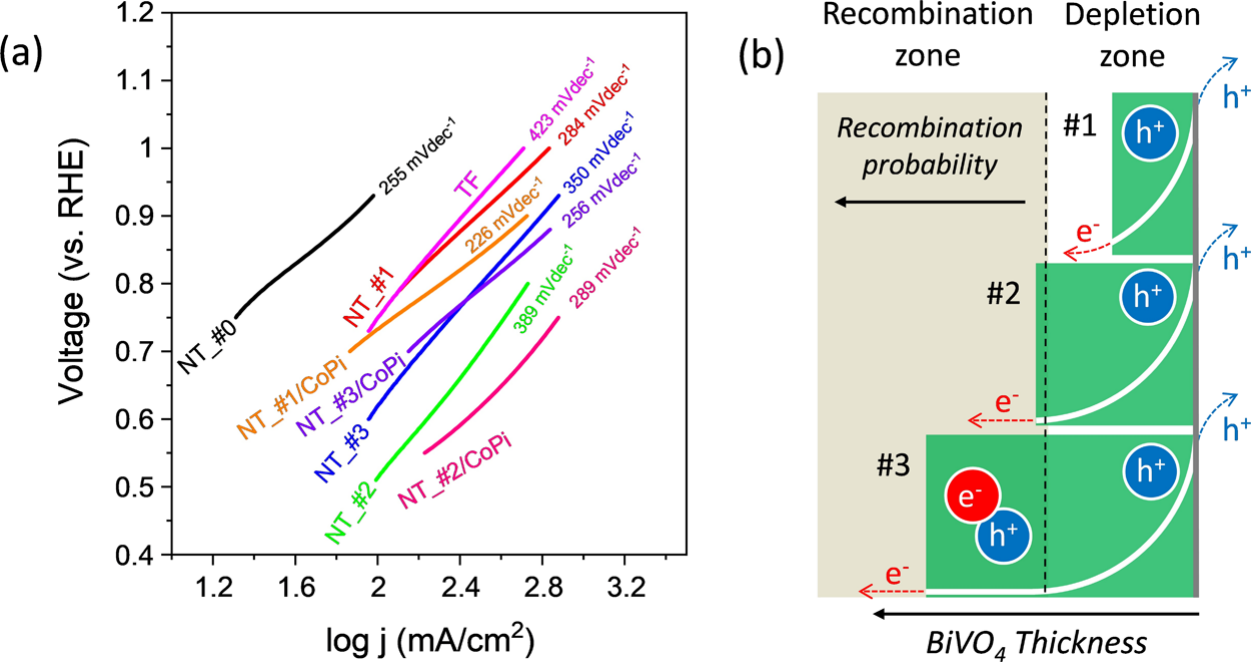
Analysis of the charge transfer process at the electrode/electrolyte interface. (a) Tafel plots for NT, NT/CoPi, and TF electrodes. (b) Band bending schemes for samples NT_#1, NT_#2, and NT_#3 taking into account an estimated thickness of BiVO_4_ layer/aggregates and how charge diffusion and recombination would be affected by the electrical field of the Schottky barrier in each case.

A Tafel analysis, based on the Butler–Volmer equation,^54^ has been also applied to estimate the kinetics of charge transfer processes at the electrode–electrolyte interface. Figure 8a shows a series of Tafel plots determined for the NT, NT-CoPi, and TF electrodes. Assuming that the majority of photoholes arriving at the surface ends up in the photooxidation of water, the slope of these plots can be taken as a measure of the kinetics of the charge transfer processes at the interface. According to this figure, slopes of electrodes NT_#0 to NT_#3 are quite similar but bigger than that determined for electrodes NT_#1/CoPi to NT_#3/CoPi, clearly indicating that the addition of the CoPi co-catalyst favors the surface reaction between photoholes and water. The Tafel slope for sample TF was higher than for NT electrodes, supporting that photoholes in this structure present a lower reactivity to generate O_2_ molecules. This result is consistent with the lower photocurrent efficiency found for the TF configuration (see Figure 4), which is also clearly related to the significantly low ECSA values depicted by sample TFs (see Supporting Information, Figure S5). It is noteworthy at this point that, despite the similar Tafel slopes for electrodes NT and NT-CoPi, log *j* at a given potential follows the order #2 > #3 > #1 (and similarly for their equivalent electrodes with CoPi). Since the photohole diffusion times determined according to eq 1 (see the previous section and Supporting Information, Figure S8) were similar for the three NT electrodes and so was the reaction kinetics of photoholes inferred by the previous Tafel slope analysis (c.f., Figure 8), the maximum efficiency found for sample NT_#2 (and NT_#2/CoPi when incorporating the co-catalyst) suggests that the number of photoholes arriving at the surface reaction sites is higher for this particular electrode.

We propose that the observed variation of efficiency with the BiVO_4_ load is due to the relation between the length of the depletion layer (i.e., the length of the Schottky barrier defined by the band bending zone) and the equivalent thickness of the BiVO_4_ sensitization layer/aggregates in samples NT_#1-NT_#3. According to the schemes in Figure 8b, the semiconductor sensitizing layer/aggregate in sample NT_#2 would have a thickness equivalent to the Schottky barrier length, thus enabling its complete development through the semiconducting layer. Under such conditions, all photogenerated charges would experience the electrical field of the band curvature enabling a more efficient separation of photogenerated electrons and holes and, consequently, diminishing photoelectron–photohole recombination probability. A similar effect was reported by us to account for the enhancement of photocatalytic activity in a rutile–anatase bilayer system.^64^ Interestingly, the average thickness determined for this layer by the statistical morphological analysis of NTs in sample NT_#2 is about 65–85 nm, within a similar range of values to that found by Selim et al.^24^ or Grigioni et al.^25^ as optimal thickness for a stable and efficient photoresponse in WO_3_/BiVO_4_ flat layer photoelectrodes. According to the scheme in Figure 8b, the equivalent thickness of the BiVO_4_ semiconductor layer/aggregates in sample NT_#1 (ca. 35 nm, see Figure 2 and Table S1) would be smaller than the charged space length, making light absorption and photoelectron/photohole separation less effective. Meanwhile, in sample NT_#3, the bigger size of BiVO_4_ aggregates (see discussion in Section 2.1) would be higher than the Schottky barrier length, making more probable the recombination of photogenerated electrons and holes in the flat zone of bands.

## Materials and Methods

### Synthesis of Multishell NT Photoelectrodes

The multishell NT electrodes were manufactured following the steps described in Scheme 1. Deposition conditions of ITO and WO_3_ layers by MS and the processing conditions of BiVO_4_ coatings and CoPi layers by, respectively, solution dripping or electrochemical methods, are detailed in Supporting Information section S1. We used a commercial glass plate covered by ITO layers as substrates (supplied by Ossila, 400 nm thickness, 15 Ω/□). ITO substrates were ultrasonically bath-cleaned following a standard sequence of acetone, ethanol, and deionized (DI) water solvents. Once in the vacuum chamber, the substrates underwent a cleaning and surface activation process by a mild radio frequency (RF) plasma treatment in an O_2_/Ar atmosphere (step (i) in Scheme 1a). Small ITO nuclei were additionally deposited by MS for very short times (step (ii)) to serve as nucleation sites for the formation of crystalline phthalocyanine ONWs (step (iii)). Details of the experimental procedure for the formation of the ONW templates by thermal evaporation can be found in previous publications^35,38,39^ (see also Supporting Information section S1). Then, ITO was deposited by MS under conditions leading to the formation of a conformal layer completely covering the ONW templates (step (iv)). The MS deposition of ITO was followed in the same chamber by the MS deposition of WO_3_ (step (v)). The steps described so far have been adopted following a one-reactor approach, i.e., using thermal evaporation and magnetron sputtering deposition following a one-reactor vacuum chamber premises. This protocol enables a rapid deposition avoiding the exposure to ambient conditions of the successively formed surfaces, as required in large-scale processes to prepare finely controlled interfaces. After vacuum deposition, ITO/WO_3_ structures were annealed in air at 450 °C for 3 h to achieve the complete removal of the organic scaffold at the core of the NTs and to induce the crystallization of the WO_3_ semiconductor oxide (step (v)).

BiVO_4_ was incorporated onto a mask delimited substrate area of 1.1 cm^2^ by sequentially dripping increasing volumes (i.e., 20, 40, 60 μL) of a solution of BiNO_3_·5H_2_O (50 mM) and VO(acac)_2_ (46.5 mM) in an acetic acid/ethyl acetate (9.5/0.5) mixture as the solvent. The dripping process was repeated up to three times to check the influence of the amount of BiVO_4_ on the photoefficiency of the electrodes. After the complete removal of the solvent (at room temperature), the samples were annealed in air at 500 °C for 4 h to induce the complete crystallization of BiVO_4_ and promote the WO_3_/BiVO_4_ heterojunction formation (see step (vi)). The final result was the hollow multishell NT structure described in Scheme 1b, step (vi), where the BiVO_4_ shell semiconductor appears externally covering the WO_3_ and ITO concentric shells. The set of fabricated WO_3_/BiVO_4_ photoelectrode samples are named NT_#0 for the ITO/WO_3_ NTs and NT_#1, NT_#2, and NT_#3 for the ITO/WO_3_/BiVO_4_ NTs. A reference flat thin film sample (TF) was prepared in the form of stacked thin films by direct MS deposition of the multilayers onto a flat ITO substrate with no ONW scaffold followed by dripping 40 mL of the BiVO_4_ precursor solution.

The cobalt phosphate (CoPi) co-catalyst was deposited using a light-stimulated electrochemical deposition method.^34^ The NT_#1-NT_#3 electrodes were back-illuminated for 600 s with the light provided by a LED 6500 K lamp (100 mW/cm^2^) in a solution of 0.15 mM Co(NO_3_)_2_·6H_2_O in a 0.1 M potassium phosphate buffer (at pH = 7.0) at a constant voltage of 0.4 V (vs Ag/AgCl (3 M KCl) reference electrode). Following the proposed notation, the CoPi set of samples has been labeled NT_#X/CoPi (X: 1, 2, and 3).

### Characterization of Multishell NT Photoelectrodes

The microstructure of the NTs was characterized by scanning electron microscopy (SEM) and transmission electron microscopy (TEM) together with EDX analysis, the latter to ascertain the layered structure of the multishell NTs. A Hitachi S4800 SEM working at 2 kV was utilized for the top-view SEM image characterization of the electrodes. Additional morphological and chemical characterization was obtained at the Helios Nanolab 650 FIB-SEM from FEI equipped with an EDX detector from Oxford (Microscopy Service from the University of Malaga). A Tecnai F30 TEM instrument working at 300 kV was utilized for the HAADF-STEM imaging, SAED, and HREM analysis.

The crystalline structure of the electrodes was ascertained by X-ray diffraction in a Panalytical X’PERT PRO model operating in the θ–2θ configuration and using the Cu Kα (1.5418 Å) radiation as the excitation source.

X-ray photoelectron spectroscopy (XPS) analysis of the outer layers of the electrodes to determine the WO_3_/BiVO_4_ coverage ratio and to confirm the chemical state of the oxide semiconductors was carried out in a PHOIBOS 100 hemispheric multichannel analyzer from SPECS, using the Al Kα radiation as the excitation source. Low- and high-resolution spectra were acquired with pass energies of 50 and 30 eV, respectively. Spectra were calibrated positioning the C1s peak at 284.5 eV.

UV–VIS–NIR absorption spectroscopy analysis was carried out recording the diffuse reflectance spectra of the electrodes with a PerkinElmer LAMBDA750S spectrometer equipped with a 60 mm diameter integrating sphere.

#### Electrochemical and Photoelectrochemical Analysis and Performance

PEC analysis of the multishell NTs and thin film TF reference electrodes was carried out in a three-electrode cell (Redox.me MM PEC 15) supplied with an illumination window (see Supporting Information, Figure S9 for details). The electrolyte was a 0.5 M Na_2_SO_4_ solution in Milli-Q DI water. Counter and reference electrodes were a 0.6 mm diameter and 250 mm long Pt wire and an Ag/AgCl (3 M KCl) commercial electrode, respectively. The electrolyte was purged with N_2_ during the experiments. All the electrochemical and PEC measurements were performed using a Metrohm Autolab PGSTAT302N potentiostat. The reference electrode voltages and the electrode current density were referred to the RHE and to the geometrical area following the conventions described in reference.^55^

The electrochemical active surface area (ECSA) of the photoelectrodes was estimated following the double-layer capacitance method.^50−53^ For that, cyclic voltammetry analysis was performed at different scan rates in the range from 2 to 50 mV/s for a voltage interval comprised between −0.05 and 0.05 mV vs Ag/AgCl (3 M KCl) reference electrode.

For the PEC characterization studies, photoanode samples were illuminated from the back side of the substrate. An Oriel Instruments 66921 arc lamp (equipped with an Oriel air mass filter AM1.5 G, reference 81094) and a Mightech PLS-6500 LED lamp were used as sun simulator and 6500 K light sources (i.e., blue region of the visible spectrum), respectively. The power flux of these two light sources was adjusted at 100 mW/cm^2^ by means of a Newport 819C-UV-2-CAL integrating sphere and a 1830R optical power meter. The 6500 K LED source provides photons within a defined range of wavelengths between 420 and 475 nm, i.e., in the spectral window between the absorption edges of BiVO_4_ and WO_3_ semiconductors (see in Figure S4 the lamp spectral distribution in relation with the diffuse reflectance spectra of the analyzed electrodes). As seen in Figure S4, the choice of the 6500 K light-emitting LED for most experiments permitted selectively exciting the BiVO_4_ semiconductor neglecting any significant contribution to photocurrent due to the direct excitation of the WO_3_ semiconductor. In addition, to conform with accepted practices and established standards of defining electrode performance and wave-length dependence of photocurrent,^55^ LSV experiments were also carried out with a standard AM1.5 G solar simulator (ORIEL 66921 arc lamp).

The IPCE response of the multishell NT electrodes was determined at 1.0 V vs RHE using a Zahner CIMPS-QE/IPCE system consisting of a tunable LED light source (TLS03). The IPCE measurements were performed in a three-electrode cell setup with a platinum foil as the counter electrode and Ag/AgCl (KCl sat.) as a reference electrode. 0.5 M Na_2_SO_4_ solution was employed as electrolyte. The IPCE values were calculated according to the following equation:
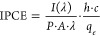
4where *I*(λ) is the photocurrent in A, *P* is the incident light power density in W/m^2^ at each wavelength λ in nm, *A* is the illuminated area in m^2^, *h* is the Planck constant (6.62607004 × 10^–34^ J s), *c* is the speed of light (∼ 3 × 10^8^ m/s), and *q_e_* is the electron charge (∼1.6 × 10^–19^ C).

Experiments to ascertain the working characteristics of the WO_3_/BiVO_4_ heterojunction as a function of the BiVO_4_ thickness were carried out by recording LSV (i.e., *i*–*V* curves between 0.5 and 1.5 V vs RHE recorded at a rate of 50 mV/s) and CA (at 1.23 V vs RHE and for periods of 3 h), under the effect of different illumination conditions with the LED source (dark, light or chopped illumination). Long-term CA experiments were performed at room temperature under the irradiation of a Mightech PLS-6500 LED light source (without UV or NIR wavelength components), while the electrolyte was continuously bubbled with N_2_. These illumination and operating conditions prevented heating of the electrolyte. Tafel plots for NT and TF electrodes, the former with and without CoPi co-catalysts, were determined according to the Butler-Volmer equation applied to the recorded LSV diagrams.^54^ EIS analysis in the dark and under illumination was carried out to estimate the characteristics of the charge transfer mechanisms during the OER. Nyquist diagrams were obtained at 1.23 V vs RHE, applying a 10 mV amplitude sinusoidal signal at frequencies varying from 10^5^ to 0.1 Hz. Fitting analysis (Metrohm Autolab NOVA 2.1.4) of these diagrams was done assuming an equivalent circuit similar to that proposed previously for this type of heterojunction electrode.^26−28^

## Conclusions

The results presented in this work confirm the optimum performance of BiVO_4_ as an effective sensitizing semiconductor photoanode in combination with WO_3_. We have found that NT electrodes, prepared using vacuum deposition procedures and ONWs as substrate-supported soft templates, present a large electrochemically active surface area available for charge transfer reactions. Furthermore, the multishell ITO/WO_3_/BiVO_4_ structure of these electrodes provides very short and equivalent charge pathways to reach the ITO conductive layer all along the complete length of the NTs. It has been also demonstrated that the thickness and homogeneity of the BiVO_4_ outer shell layer is an additional parameter, which optimized to a value in the range of 65–85 nm, and permits maximization of the cell photocurrent.

Within the range of studied photon fluxes, the WO_3_/BiVO_4_ heterojunction structure of the NT electrodes did not present signs of saturation, a feature enabling the use of these electrodes under very high illumination conditions as those provided by solar collector devices. The robust character of the system (it could be reused without any noticeable loss of activity) and its long time stability make these electrodes a suitable option for practical applications. This capacity is further justified by the scalability of the processing methods utilized for their fabrication. Key issues by the utilized synthesis procedure are the use of vacuum processable ONWs as a scaffold and the demonstration that the MS technique can be used for the preparation of conformal layers of ITO and WO_3_ to completely cover 10 μm-long NWs. We have also shown that the hollow multishell NT configuration is perfectly compatible with the incorporation of the OER co-catalyst. This has been proven by the incorporation of a CoPi layer and the demonstration of an enhancement in PEC efficiency with photocurrents of 2.23 mA/cm^2^ at 1.23 V vs RHE under 1 sun illumination. This value, although not maximal,^22^ is within the average of most recent works in the literature.^10,65^ It is expected that thanks to the fine control of the multishell composition, the actual electrochemically active surface area and nanostructure of NTs achieved with the applied synthesis procedure, and adapting the co-catalyst application procedure, the PEC efficiency of the cells can be further enhanced.

## Supporting Information Available

Experimental details for the NT catalyst system synthetic protocol (Section 1); thicknesses, mean value and standard deviation data for a selected set of NTs (Table S1); FIB cross-section images of selected samples (Figure S1); SEM micrographs of the TF configuration sample (Figure S2); XPS and EDX complementary analysis (Figure S3); UV-Vis diffuse reflectance characterization of NTs samples (Figure S5); ECSA measurements (Figure S5); PEC complementary analysis (Figure S6); comparative photocurrent values extracted from LSV and CA measurements (Figure S7); PEC transient analysis (Figure S8); and experimental setup used in this work (Figure S9) (PDF)
