# Supplementary material for: Photoelectrochemical Water Splitting with ITO/WO3/BiVO4/CoPi Multishell Nanotubes Enabled by a Vacuum and Plasma Soft-Template Synthesis
Source: ACS Appl Mater Interfaces. 2023 Feb 10;15(7):9250–62. doi: 10.1021/acsami.2c19868 (PMC9951206; doi:10.1021/acsami.2c19868)
Supplement: Supplementary file 1 — am2c19868_si_001.pdf [file am2c19868_si_001.pdf]

SUPPORTING INFORMATION

Photoelectrochemical water splitting with ITO/WO_3/$BiVO_4$/CoPi multishell nanotubes enabled by a vacuum and plasma soft-template synthesis

Jorge Gil-Rostra [1]; Javier Castillo-Seoane [1]; Qian Guo [2]; Ana Belén Jorge Sobrido [2]; Agustín R. González-Elipe [1]; Ana Borrás [1]*

(1) Nanotechnology on Surfaces and Plasma Lab, Instituto de Ciencia de Materiales de Sevilla (CSIC-US). Avenida de Américo Vespucio, 49, 41092, Sevilla, SPAIN.

(2) School of Engineering and Materials Science, Queen Mary University of London, E1 4NS, London, UK

*Corresponding author: jorge.gil@icmse.csic.es

Section S1. Multishell NTs synthesis protocol: experimental details.

(o) Substrates cleaning process:

Commercial glass plates (25x25 mm) covered by ITO layers (supplied by Ossila, 400 nm thickness, 15 Ω/□) were used as substrates. Samples deposited on fused silica slides and single crystalline P-type doped (100) silicon wafers were also used for complementary structural and physicochemical characterization. All substrates were sonicated following standard acetone (Sigma Aldrich, ACS reagent) 15 min, ethanol (Sigma Aldrich, absolute grade) 15 min, and deionized water (Milli-Q) 15 min, solvents sequence.

(i) Radio frequency cleaning and superficial activation process:

Once in the vacuum chamber, the substrates underwent a plasma cleaning and surface activation process. A radio frequency (RF) plasma was ignited directly on the chamber's substrate holder using a Hunttinger PFG300 RF generator powered at 50 W (DC bias 225 V) in an atmosphere of 50% O_2/Ar for 15 minutes. Gases were dosed using mass flow controllers (Bronkhorst, Ar: 15 cm^3/min, O_2: 15 cm^3/min). The chamber working pressure was $5.0x10^{-3}$ mbar.

(ii) ITO nucleation layer deposition process:

A very thin film of ITO (25 nm) was deposited acting as a nucleation layer to facilitate the crystalline formation of the organic phthalocyanine nanowires. A cylindrical MS head (AJA International Inc.) equipped with a 3” ITO disc target (In_2O_3/SnO_2 90/10, Testbourne Ltd.) was powered at 75 W using a Hunttinger PFG300 RF source in Ar atmosphere (Bronkhorst, Ar: 30 cm^3/min). The pressure was regulated using a butterfly valve and was controlled using a Pirani pressure meter. The working pressure was kept at $5.0x10^{-3}$ mbar. The substrate holder to MS source distance was 15 cm. The substrate holder temperature was controlled at 350 ˚C with a spinning velocity of 20 rpm. The deposition rate and thickness during de deposition process were registered with a quartz crystal microbalance monitor placed close to the sample holder.

<u>(iii) Phthalocyanine (H2Pc) organic nanowires:</u>

Organic nanowires (ONWs) were grown by thermal evaporation using a LUXEL Radak thermal evaporator loaded with 29H, 31H-Phthalocyanine (Sigma Aldrich, 98%). The alumina crucible was heated from 350 °C to 375 °C to maintain a uniform H2Pc flux and a constant deposition rate. The thermal evaporator was operated with a PID power controller and the deposition rate was registered with a quartz crystal microbalance monitor. The deposition process was carried out in Ar (Bronkhorst, Ar: 10 cm^3/min). The working pressure was $1.5x10^{-3}$ mbar. The substrate holder

temperature was set at 210 °C with a spinning velocity of 20 rpm. In these conditions, the H_2Pc deposition rate was approximately 0.15 Å/s. The evaporation process was maintained until reaching an organic precursor nominal thickness of 0.35 kÅ.

(iv) ITO magnetron sputtering deposition process:

ITO was deposited by MS under operating conditions leading to a conformal layer completely covering the previously deposited ONW nanostructured templates. The deposition conditions were similar to those described in step (*ii*). To protect the ONW structure the substrate holder temperature was set at 210 °C during the initial stages of the deposition. After 10 minutes, the holder temperature was raised to 350 °C and was kept constant until the end of the deposition process. The ITO layer's nominal thickness was 250 nm.

(v) WO_3 magnetron sputtering deposition process:

WO_3 films were deposited by reactive MS. A cylindrical MS head (AJA International Inc.) equipped with a 3" tungsten disc target (Testbourne Ltd., 99%) was powered at 150 W using a DC

pulsed (120 kHz) Advanced Energy Pinnacle + source in an atmosphere of 50% Ar/O_2 (Bronkhorst, Ar: 15 cm^3/min, O2. 15 cm^3/min). The working pressure was $5.0x10^{-3}$ mbar. The substrate holder temperature was set at 350 ˚C with a spinning velocity of 20 rpm. The process parameters (pressure, gas flow, film thickness, temperature, etc.) were adjusted by the control elements previously described in sections (*i*) to (*v*). The final WO_3 layer's nominal thickness was 500 nm.

After vacuum deposition, ONW/ITO/WO_3 structures were annealed in air at 450 °C (2 °C/min) for 3 h until the complete removal of the ONWs at the core of the NTs.

(vi) $BiVO_4$ drop-casting deposition:

$BiVO_4$ was incorporated by sequentially dripping on a delimited substrate area of 1.1 cm^2 different volumes (i.e. 20, 40, 60 µl) of a solution of $BiNO_3x5H_2O$ (Sigma Aldrich, 99.99%) (50 mM), $VO(acac)_2$ (Sigma Aldrich, 98%) (46.5 mM) and an acetic acid/ethyl acetate (9.5/0.5) mixture as solvent. Before use, the solution was agitated and periodically sonicated for 5 hours. After the complete removal of the solvent (at room temperature) the samples were annealed in air at 500 °C (2 °C/min) for 4 h.

(vii) Cobalt phosphate light stimulated electro-deposition:

The cobalt phosphate (CoPi) co-catalyst was deposited through a light-stimulated electrochemical deposition method. For that, the electrodes were back-illuminated with the light provided by a LED 6500K lamp at 100 mW/cm^2, in a solution of 0.15 mM $Co(NO_3)_2x6H_2O$ (Sigma Aldrich, 99.99% trace metals basis) in a 0.1 M potassium phosphate buffer (at pH=7.0) applying a constant voltage of 0.4 V (vs. Ag/AgCl (3M KCl) reference electrode) for 600 s.

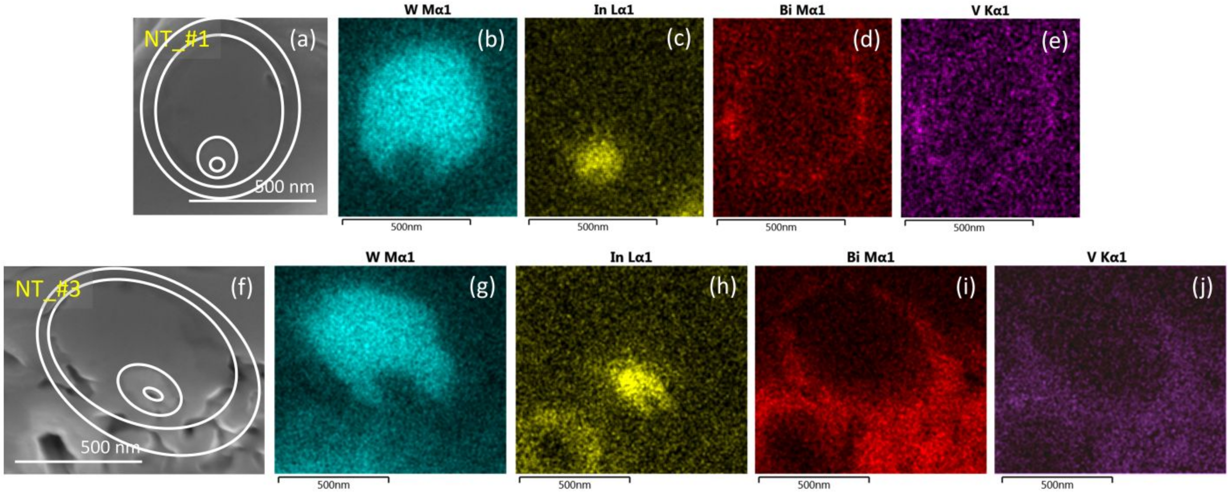

Figure S1. *FIB cross-section of two different nanotubes extracted from NT_#1 (a) and NT_#3 (f) and corresponding EDX mapping for representative elements W (b-g), In (c-h), Bi (d-i) and V (e-j).*

Table S1. *Thicknesses (x10), mean value and standard deviation (SD) data measured from SEM micrographs for a selected set of representative nanotubes of samples NT_#0 to NT_#3.*

											**Mean**	**SD**
**NT_#0**	471	399	359	457	524	351	386	424	475	329	**417**	63
**NT_#1**	536	473	455	504	520	460	455	478	431	486	**480**	32
**NT_#2**	565	561	555	504	508	601	576	472	614	546	**550**	44
**NT_#3**	604	558	505	595	599	530	620	615	577	646	**585**	43

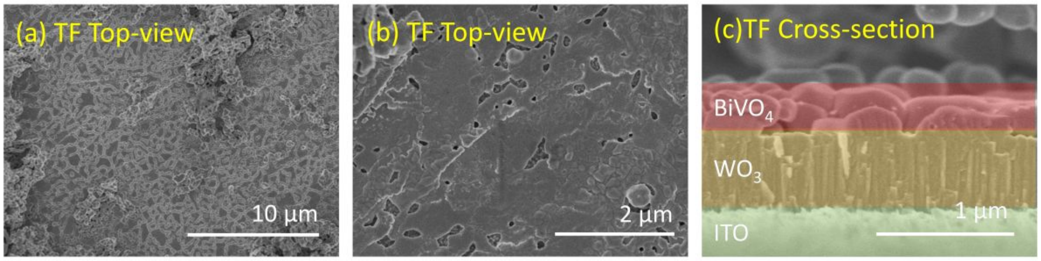

Figure S2. *SEM micrographs for top-views (a-b) and cross-sections (c) (colourful for easier visualization of the layers) for the thin film configuration (sample TF) with an equivalent* $BiVO_4$ *thickness to sample NT_#2.*

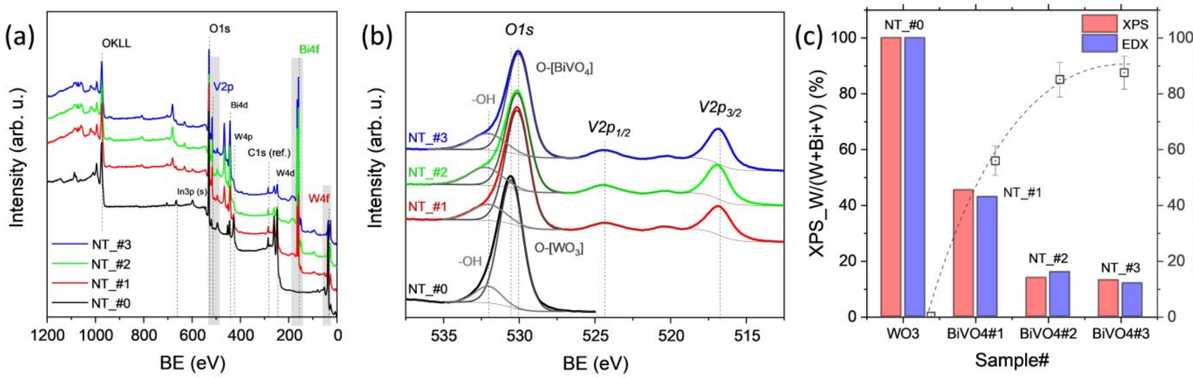

Figure S3. *(a) XPS low-resolution spectra of samples NT_#0 to NT_#3. W4f, Bi4f and V2p photoemission signals have been highlighted. (b) XPS O1s and V2p zone high-resolution spectra. O1s signals have been deconvoluted to show their (O-M) and (-OH) components. (c) XPS and EDX surface quantification of the W atomic ratio and the $BiVO_4$ degree of coverage for samples NT_#0 to NT_#3 as a function of the number of additions of the $BiVO_4$ precursor solution.*

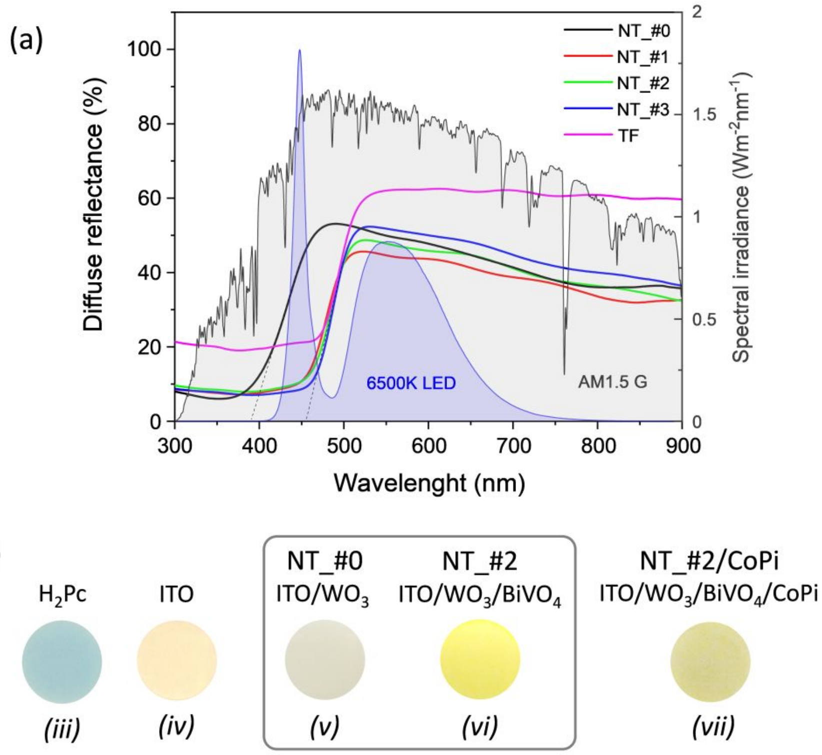

Figure S4. *(a) Diffuse reflectance spectra of samples NT_#0-#3 and sample. Superimposed and for comparative purposes, spectral distribution of LED 6500K and AM1.5 G light sources used in this work. (b) Colour evolution (actual photographs) of the in-process samples through the different steps of the photo-electrode fabrication. Phthalocyanine organic nanowires (iii), ITO nanotubes (iv), ITO/*WO_3 *NTs, ITO/*WO_3*/*$BiVO_4$ *NTs (vi), and ITO/*WO_3*/*$BiVO_4$*/CoPi NTs (vii).*

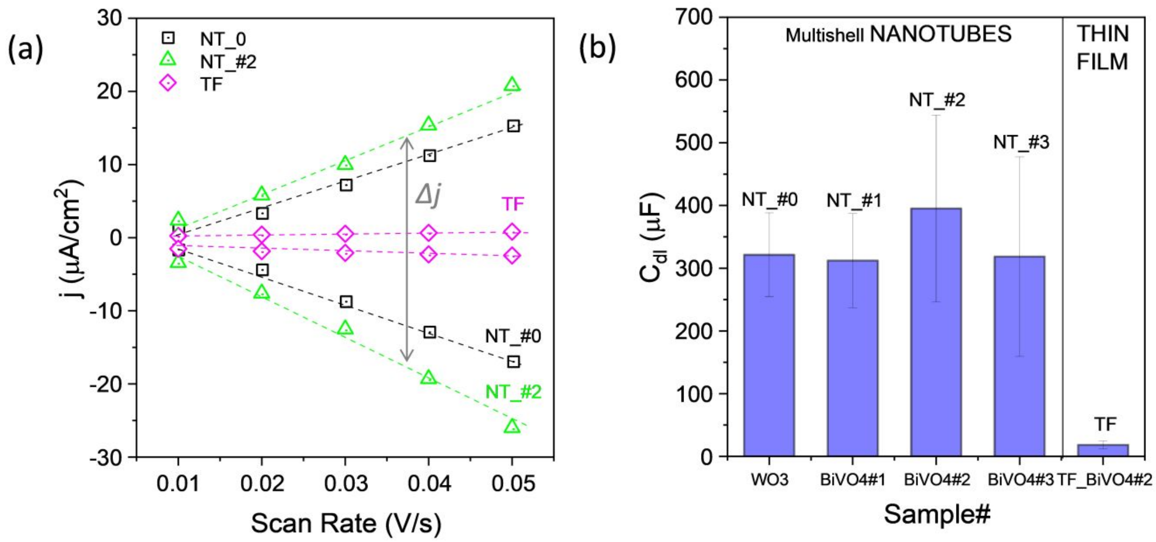

$$ECSA = \frac{C_{dl}}{C_s} \quad C_{dl} = \frac{d(\Delta j)}{2d(SR)} \ (1)$$

Figure S5. *(a) Anodic and cathodic current density values extracted from ECSA measurements (CV from -0.05 to 0.05 mV vs. Ag/AgCl (3 M KCl)) vs. scan rate (SR) for a selected set of samples NT_#0, NT_#2, and TF_#2. (b) Column diagram of the calculated double layer capacitance (C_{dl}) corresponding to NT_#0-#3 and TF samples.*

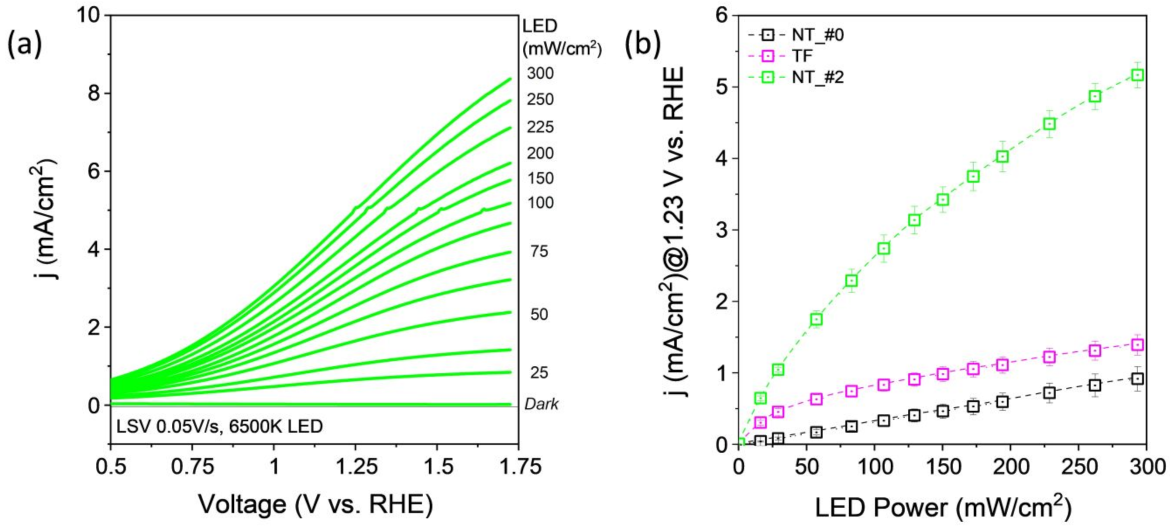

Figure S6. (a) Photoelectrochemical LSV curves measured for sample NT_#2 recorded under LED 6500K illumination at different power intensities. (b) Photocurrent values determined for samples NT_#0, NT_#2 and TF as a function of light power intensity (LED 6500K) for a fixed sample polarization voltage of 1.23 V vs. RHE.

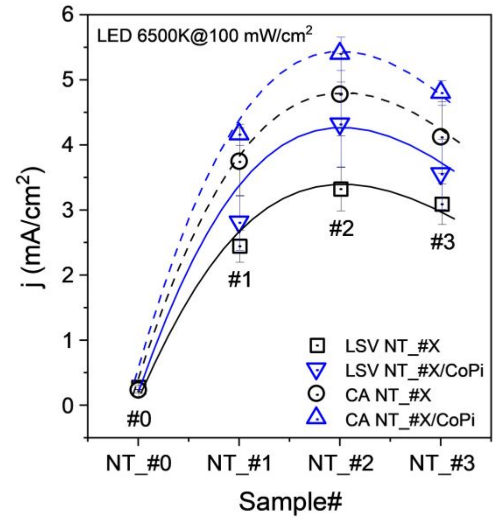

Figure S7. *Comparative photocurrent values measured at 1.23 V vs. RHE under 100 mW/cm² LED 6500K illumination power from LSV and CA (mean value) photoelectrochemical measurements corresponding to NT_#0-#3 and NT_#1/CoPi-#3/CoPi sample series.*

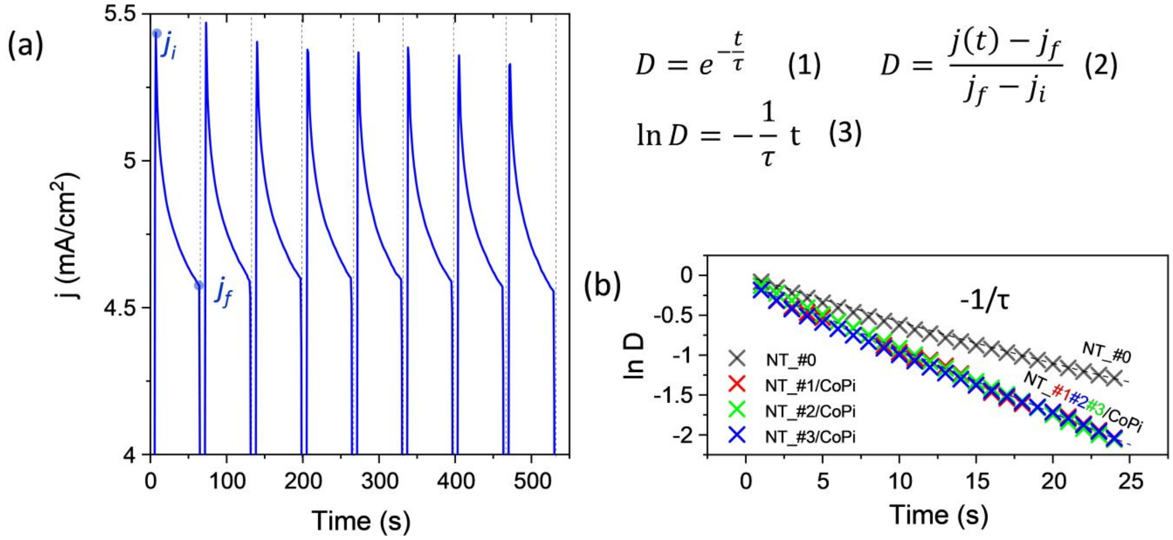

Figure S8. *(a) Photoelectrochemical CA response of sample NT_#2 measured under chopped illumination (LED 6500K light source at 100 mW/cm^2) at 1.23 V vs. RHE. The reference parameters j_i and j_f, defined for equation (2) as the current density at the beginning and the end of the transient, are highlighted in the figure. (b) ln D vs. t representation for samples NT_#0 and NT_#1/CoPi-#3/CoPi (b) As indicated in equation (3), the transient diffusion time (τ) can be estimated from the slope of the linear fitting of the data.*

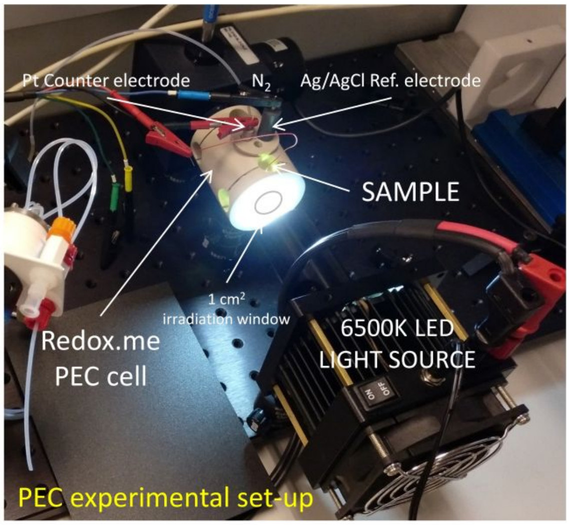

Figure S9. *Experimental set-up used in this work for the photoelectrochemical characterization of multishell nanotubes. The system consisted of three electrodes photoelectrochemical cell (Redox.me MM PEC 15) and a 6500K LED light source (Mightech PLS-6500) or a solar radiation simulator (Oriel Instruments 66921 arc lamp, not shown here).*
